# Sensory-emotional-cognitive effects of resistance exercise and Tai Chi exercise in Japanese community-dwelling older adults with chronic pain: a non-randomized controlled trial

**DOI:** 10.1186/s12906-025-05100-9

**Published:** 2025-10-10

**Authors:** Cen Chen, Takafumi Saito, Lefei Wang, Harukaze Yatsugi, Ziming Gong, Sitong Li, Hiro Kishimoto

**Affiliations:** 1 Department of Behavior and Health Sciences, Graduate School of Human- Environment Studies, Kyushu University, Fukuoka, 819-0395 Japan; 2 Faculty of Rehabilitation, School of Physical Therapy, Reiwa Health Sciences University, Fukuoka, 811-0213 Japan; 3 Faculty of Arts and Science, Kyushu University, IC15, 744 Motooka, Nishi-ku, Fukuoka, 819-0395 Japan; 4 Center for Health Science and Counseling, Kyushu University, Fukuoka, 819-0395 Japan

**Keywords:** Chronic pain, Older adult, Tai chi

## Abstract

**Background:**

Although Tai Chi has demonstrated efficacy in alleviating pain and improving functional mobility in people with chronic pain, the mechanisms underlying its pain-relieving effects remain poorly understood. We assessed the efficacy of a Tai Chi intervention on pain-related sensory, emotional, and cognitive indices in Japanese community-dwelling older adults with chronic pain.

**Methods:**

A non-randomized controlled trial was conducted for 12 weeks in the community of Itoshima City, Fukuoka, Japan. Adults aged ≥ 60 years with chronic pain were recruited and allocated to an intervention group or a control group. A total of 84 participants were allocated, with 44 in the intervention group and 40 in the control group. Chronic pain was defined as musculoskeletal pain lasting ≥ 3 months. Baseline characteristics of participants included age, sex, education, body mass index, comorbidities, current tobacco consumption, current alcohol consumption, and fall history. The control group received resistance training; the intervention group received resistance training and Tai Chi exercise. The main outcomes were assessed at baseline and after 12 weeks of intervention: a pain numeric rating scale (NRS), the pressure pain threshold (PPT), the Tampa Scale of Kinesiophobia (TSK), the Pain Catastrophizing Scale (PCS), and the Central Sensitization Inventory (CSI). As secondary outcomes, TSK and PCS subscales were evaluated with the same procedures. The statistical analyses used the t-test, χ^2^-test, Wilcoxon rank-sum test, and analysis of covariance with adjustment for baseline characteristics.

**Results:**

No baseline characteristics differed significantly between the groups. At 12 weeks, the intervention group showed significant improvements in the NRS (*p* = 0.02, 95%CI: −2.20, − 0.18), PPT (*p* = 0.003, 95%CI: 0.22, 0.99), TSK (*p* = 0.004, 95%CI: −6.35, − 1.22), and PCS (*p* = 0.01, 95%CI: −10.18, − 1.43) versus the control group. There was no significant between-group difference in the CSI values. After adjustment for baseline characteristics, significant interactions were revealed between time and group for PPT (*p* = 0.02), TSK (*p* = 0.02), and PCS (*p* = 0.03) as well as the subscales TSK-Somatic Focus (*p* = 0.02) and PCS-Rumination (*p* = 0.01).

**Conclusion:**

Tai Chi intervention may serve as a potential treatment for chronic pain by addressing sensory, emotional, and cognitive aspects of pain.

**Trial registration:**

The University Hospital Medical Information Network Clinical Trials Registry, UMIN000052727, Date: 2023-11-13. https://center6.umin.ac.jp/cgi-open-bin/ctr_e/ctr_view.cgi?recptno=R000058307.

## Introduction

Chronic pain has been shown to be associated with poor health outcomes [1], affecting over 30% of people worldwide [2], with aging being a significant contributor to its onset [3]. According to findings from the Japan Gerontological Evaluation Study which examined a cohort of older adults in Japan, the prevalence of chronic pain among the independent older adults was 39.0% [4]. As the population of individuals at advanced ages in Japan continues to expand, it is anticipated that the prevalence of chronic pain among older Japanese will rise, leading to widespread disability, diminished quality of life, and substantial burdens on the healthcare system [5].

Chronic pain is a complex condition defined as pain persisting beyond the usual course of acute illness or injury, typically lasting ≥ 3 months [6]. It is often aggravated by a mechanism of central sensitization against a background of biopsychosocial factors such as emotional (e.g., fear of movement) and problematic cognitive factors (e.g., catastrophic thinking) [7]. Common management strategies for chronic pain typically include pharmacological interventions such as analgesics, anti-inflammatory drugs, and opioids, as well as non-pharmacological approaches. Medications are often effective in the short term but may have limited efficacy or significant side effects with long-term use [8].

In recent years, there has been an emphasis on the nonpharmacologic management of chronic pain, such as mind-body exercise [9]. Mind-body exercise may prove to be more beneficial for older adults living with chronic pain due to its multi-modal meditative component incorporated into physical movement [10]. Tai Chi, as a mind-body exercise and multicomponent intervention, combines slow, flowing movements with breathing adjustment, relaxation techniques, and meditation. Evidence suggests that Tai Chi may have positive treatment effects on patient outcomes, including hypertension, the prevention of falls, cognitive performance, depression, balance confidence, and muscle strength, suggesting its potential as a non-pharmacological intervention to prevent the deterioration of health and improve many situations that lead to excessive medical utilization [11].

Although the potential limitations of Tai Chi exercises have been reported to be a barrier for those with limited mobility and time [12], the therapeutic effects of Tai Chi on chronic pain have become well known worldwide, with guidelines from the American College of Physicians and the UK National Guideline Centre recommending Tai Chi as a treatment option [13, 14]. Despite the growing body of research on the effectiveness of Tai Chi in managing chronic pain, the mechanisms that underlie this effectiveness have yet to be rigorously evaluated and thus remain unconfirmed. It is plausible that for musculoskeletal conditions, the mechanisms may be largely behavioral in nature, suggesting that Tai Chi might influence pain outcomes through changes in behavior, such as increased physical activity and improved movement patterns [15]. Given this potential for behavioral change, it is essential to focus on the emotional and cognitive aspects that influence these behaviors. In addition, central sensitization also plays a significant role in the mechanism of chronic pain, characterized by an increased response to pain stimuli and widespread pain hypersensitivity [16].

Considering that chronic pain involves multidimensional aspects beyond pain intensity and functional disability, the necessary research should incorporate multiple assessments such as sensory aspects (the pressure pain threshold and central sensitization), emotional factors (fear avoidance), and cognitive aspects (catastrophic thinking), which might shed some light on the mechanisms underlying the behavioral changes and pain-relieving effects of Tai Chi as a multimodal therapy. However, our literature search identified no published examinations of the adherence and effectiveness of Tai Chi in Japanese community-dwelling older adults with chronic pain from a multidimensional perspective. This gap in the literature highlights the clinical significance of our study, which offers novel insights into non-pharmacological treatments for chronic pain. Our study may support the efficacy of Tai Chi in pain management, inform evidence-based clinical strategies, and enhance patient awareness of the emotional and cognitive dimensions of chronic pain.

To address this gap, we conducted the present study to (*i*) assess the feasibility and acceptability of both Tai Chi and resistance training performed for 12 weeks by Japanese community-dwelling older adults with chronic pain, and (*ii*) investigate the sensory-emotional-cognitive effects of Tai Chi on chronic pain. We hypothesized that Tai Chi exercises could achieve a pain-relief effect through improvements of the pressure pain threshold, kinesiophobia, pain-catastrophizing, and central sensitization in older adults with chronic pain.

## Subjects and methods

### Study design and participants

We conducted a non-randomized controlled intervention study consisting of control and intervention groups followed for 3 months (12 weeks). All participants were recruited from Itoshima City, Fukuoka through community flyers and posters. The inclusion criteria for participants were: (1) adults aged ≥ 60 years; (2) reported musculoskeletal pain at least one site; (3) pain symptoms for ≥ 3 months; (4) not involved in any Tai Chi exercise program in the past 6 months; and (5) having the capability to independently ambulate and communicate well. The exclusion criteria were: (1) diagnosis of a disease/condition that would interfere with study participation (e.g., cancer, Parkinson’s disease, or psychiatric disorder); and (2) requiring assistance or caregiving in their daily lives. All participants provided written informed consent. The study was approved by the Institutional Kyushu University Health Sciences and Counseling Center Joint Ethics Committee, Japan (approval no. 202306) and registered in the University Hospital Medical Information Network Clinical Trials Registry (UMIN000052727).

### Sample size

According to a study demonstrating significant effects of Tai Chi on participants’ pain intensity, the effect size (d) of pain intensity scores after 12 weeks of intervention was 0.83 points [17]. To detect a similar effect size of 0.83 with a two-tailed significance level of 0.05 and a power of 80%, G*Power (ver. 3.1) calculated a required sample size of 24 participants for one group. Since we compared two groups, a total of 48 participants was necessary. Considering a dropout rate of 20% based on similar studies [18, 19], 60 participants needed to be recruited at baseline.

### Intervention

Participants’ safety and well-being were closely monitored throughout the study. Regular check-ins were conducted to address any discomfort, and stringent safety protocols were maintained, including supervision by trained personnel and adherence to standardized warm-up and cool-down procedures.

#### The control group

The control group participated in a 12-week resistance training program (60 min per session, 1×/week). The resistance training focused on the use of resistance bands (e.g., TheraBands), improvised weights such as plastic bottles filled with water (as substitutes for dumbbells), and body weight exercises. Each session was conducted in a group format at a local community center, with a 5-min warm-up, 45-min resistance training practice, 5-min break, and cooling down for 5 min. The sessions were supervised by three qualified Health Fitness Programmers (certified by the Japan Health Exercise and Fitness Foundation) who paid close attention to the participants and their needs, helping them master their psychological and emotional changes. The participants were instructed to continue their usual activities and refrain from joining any new exercise classes during the 12-week intervention period.

#### The intervention group

In addition to the resistance-training regimen described above, the intervention group practiced Yang-style 24-form Tai Chi in a 60-min session 1×/week for 12 weeks, led by a professional Tai Chi instructor who had 2 years of teaching experience. In the first session, the participants received printed materials on Tai Chi principles, practice techniques, and safety precautions. The instructor explained the Tai Chi exercise theory (the philosophy of yin and yang) and procedures. The whole set of Tai Chi included a 5-min warm-up, 45-min Tai Chi practice, a 5-min break, and 5 min for cooling down.

During the Tai Chi practice, emphasis was placed on the transfer of the body’s center of gravity and multi-segment movement coordination of the trunk, incorporating characteristic abdominal breathing as part of the exercise, and integrating it into the practice. The abdominal breathing training included the traditional Chinese Qi Gong technique ‘qi cheng dantian’ (for qi to sink to the lower dantian). The lower dantian is located in the lower abdomen, approximately three finger-widths below the navel and in the center of the body. The participants were instructed to focus on inhaling slowly and deeply into the lower abdomen to nourish and strengthen the lower dantian, promoting relaxation, stability, and the smooth flow of qi throughout the body in the Tai Chi practice.

### Measurements

The assessments, including validated questionnaires and physical evaluations, were carried out by a multidisciplinary team comprising physician, nurse, physical therapists, and trained staff. To reduce the risk of bias, a blinding procedure was implemented during the outcome-assessment phase. The individuals responsible for assessing the outcomes were blinded to the participants’ group allocation and were not involved in the application of the intervention.

#### Feasibility and acceptability

The feasibility and acceptability of the two interventions were based on how successfully the Tai Chi and resistance-training interventions were implemented. The evaluation was based on the number of study participants recruited, the retention rate, and the adherence rate within each group. Attendance was documented for each exercise session, with an instructor-led session attendance rate < 20% considered non-adherence [19, 20].

#### Assessment of pain characteristics

Chronic pain was assessed using questions ascertaining the respondent’s pain lasting ≥ 3 months in the prior 12 months [6]. We asked, ‘Have you had any areas of the body where pain has persisted for 3 months or longer in the past year?’ [21] They could respond with a “yes” or “no.” If they answered “yes,” they were further prompted to indicate the specific musculoskeletal area(s) affected, including the neck, shoulders, elbows, wrists and/or hands, hips, knees, feet, and low back, using a body diagram. The subtypes of chronic pain were assessed by considering the number of pain sites and pain intensity. A subjective numerical rating scale (NRS) was provided to the participants for the evaluation of pain intensity (0–10 points with 0 representing no pain and 10 representing the worst pain) [22]. The NRS is widely used for pain assessment, and its reliability and validity for evaluating chronic pain have been confirmed [22]. Pain intensity was measured before the intervention (baseline) and at 12 weeks post-intervention.

#### Pain-pressure threshold (PPT)

The measurements of the pain-pressure threshold (PPT) were conducted using an algometer (NEUTONE TAM-Z2 (BT10); TRY-ALL, Chiba, Japan). The algometer is widely used for assessing individuals’ PPT and has demonstrated strong reliability and validity [23]. With continuously increasing pressure, the soft tissue in the measurement area was compressed with the metal rod of the algometer. The PPT test stimulus was applied to the participant’s reported most painful site. After the 12 weeks of intervention, PPT measurements were taken again at the same painful site. The participants were asked to say “pain” when the sensation of pressure or discomfort became a clear sensation of pain. The value read from the device at this time point (kilograms per square centimeter) corresponded to the PPT. Three measurements were collected for each site at 30-sec intervals. The average of the three measurements was used for the data analysis [23]. The participants’ PPT was evaluated before the intervention (baseline) and at 12 weeks post-intervention.

#### Tampa scale of kinesiophobia (TSK)

The Tampa Scale of Kinesiophobia (TSK) has been used to distinguish between non-excessive fear and phobia in individuals with chronic musculoskeletal pain. The TSK scale consists of 17 items, with participants rating their fear of movement causing pain and injury on a scale from 1 to 4, resulting in scores ranging from 17 to 68. The TSK includes two subscales: activity avoidance (TSK-AA), reflecting the belief that activity may lead to (re)injury or increased pain, and somatic focus (TSK-SF), indicating the belief that pain signifies underlying and serious medical issues [24]. The Japanese version of the TSK has been demonstrated to be psychometrically reliable and valid for detecting fear of movement [25]. In the present study, the TSK was administered before the intervention (baseline) and at 12 weeks post-intervention.

#### Pain catastrophizing scale (PCS)

The Pain Catastrophizing Scale (PCS) is a 13-item self-report measure designed to assess catastrophic thinking related to pain among adults with chronic pain. It is scored from 0 to 4, resulting in a total score ranging from 0 to 52. The PCS scale consists of three subcategories: rumination (PCS-R), helplessness (PCS-H), and magnification (PCS-M), whose added scores provide the total PCS score [26]. The Japanese version of the PCS has been confirmed to have valid reliability and validation [27]. In the present investigation, the PCS was administered before the intervention (baseline) and at 12 weeks post-intervention.

#### Central sensitization inventory (CSI)

Many chronic musculoskeletal pain conditions are characterized by hypersensitivity, which is induced by central sensitization. The Central Sensitization Inventory (CSI) was designed to identify individuals whose presenting symptoms may be associated with central sensitivity syndrome (CSS). The CSI score ranges from 0 to 100, comprising 25 self-reported questions rated from 0 to 4 points. The questions address various components of downstream symptoms, including feelings of depression or low energy, muscle stiffness, poor sleep, and light sensitivity. A higher score indicates greater symptom severity, potentially indirectly linked to conditions involving underlying central sensitization [28]. The Japanese version of the CSI was reported to be a reliable and valid tool to assess CSS in Japanese patients with musculoskeletal disorders [29]. We administered the CSI before the present intervention (baseline) and at 12 weeks post-intervention.

#### Demographic and health characteristics

Socio-demographic characteristics including age, sex, education level (< 10 vs. ≥10 yrs), comorbidities (including at least one of the following diseases: osteoporosis, hypertension, hyperlipidemia, diabetes, heart disease, osteoarthritis, stroke, rheumatism, herniated disc, and spinal stenosis), current tobacco consumption, current alcohol consumption, and fall history in the past 12 months were also collected via a questionnaire. Each participant’s body weight (kg) and body height (cm) were measured using standard protocols with the participant in light clothing and without shoes. The participants’ body mass index (BMI) values were calculated as weight in kg divided by height in m^2^.

We used the Pain Disability Assessment Scale (PDAS) to evaluate pain-related disability. The PDAS is a 20-item tool that assesses the degree of impact of pain-related disability on a person’s activities of daily living (ADLs) during the past week. Respondents are asked to rate each activity on a Likert scale from 0 (“Pain did not interfere with this activity”) to 3 (“Pain completely interfered with this activity”). Total scores range from 0 to 60 points, with higher scores indicating greater levels of pain interference [30].

### Statistical analyses

Descriptive data are presented as the mean (± standard deviation [SD]) and median (interquartile range) for continuous variables and as the frequency (percentage) for categorical variables. The t-test and the Wilcoxon rank-sum test for continuous data and the χ^2^-test for categorical data were used to compare baseline differences between the intervention group and the control group. Paired t-tests were used to compare outcome measures before and after the 12-week exercise interventions within each group. Independent t-tests were used to compare the pre- to post-intervention differences between the two groups. The 95% confidence intervals (CIs) were calculated to provide a measure of the precision of the estimates. To investigate intervention effects between the groups, we compared differences in changes across the two arms by conducting an analysis of covariance (ANCOVA) with adjustment for baseline characteristics (age, sex, education, BMI, comorbidities, current tobacco consumption, current alcohol consumption, fall history; the time × group interaction effects across the baseline and 12-week intervention period were then examined.

Cohen’s d-values were calculated to quantify the effect size. We also used the widely accepted method of 0.5 of the standard deviation to calculate the minimal clinically important difference (MCID) [31]. This method states that a change of ≥ 0.5 of the standard deviation is the smallest clinically significant change in scores from baseline to follow-up [31]. SPSS software (ver. 21.0; IBM, Armonk, NY, USA) was used for the statistical analyses. Statistical significance was defined as a two-sided p-value < 0.05.

## Results

**Fig. 1:**
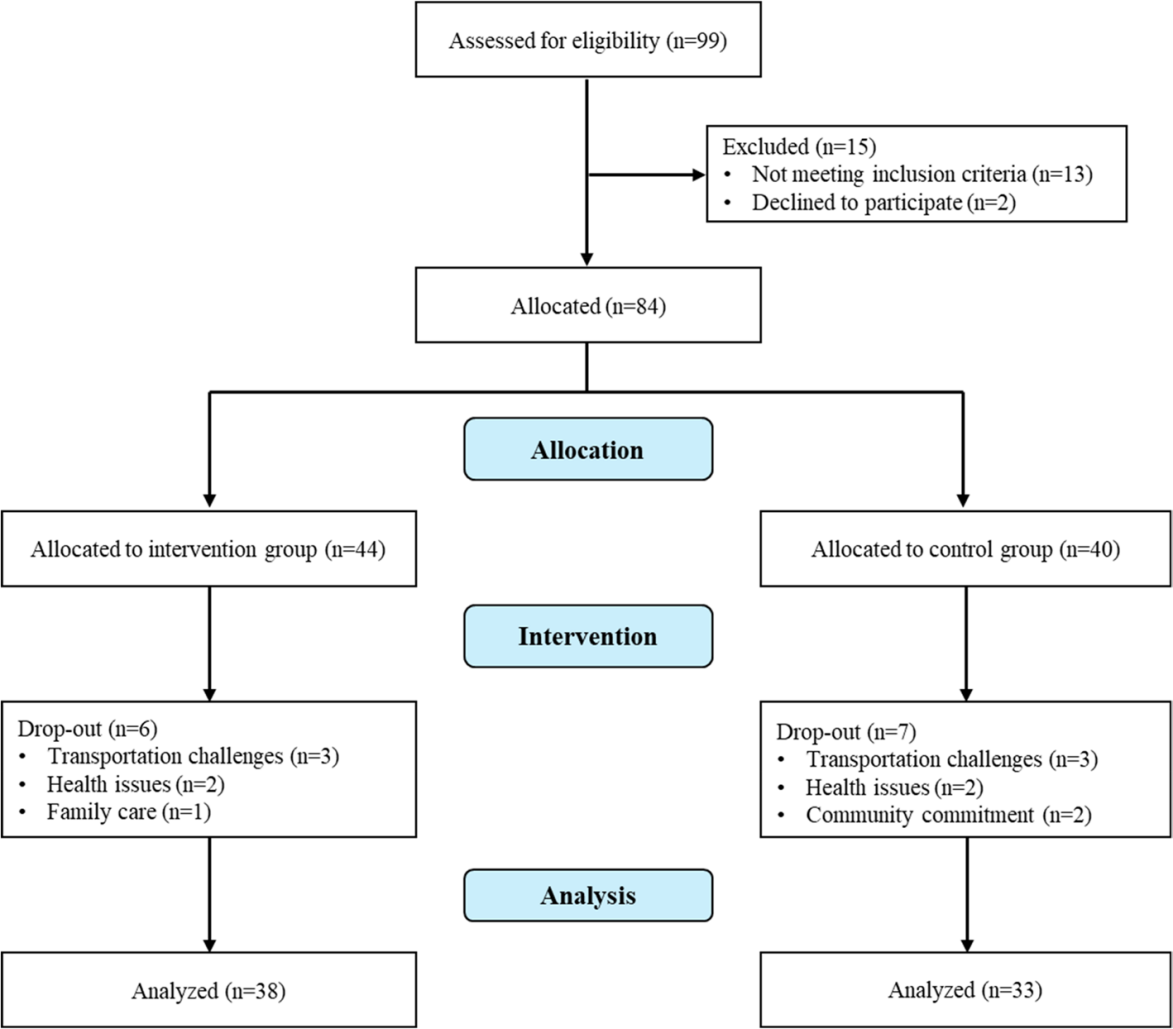
Flow diagram of study participation

Our original enrollment target was 60 participants with an anticipated dropout rate of 20%. Of the 99 older adults screened, 13 did not meet the inclusion criteria and two declined to participate. The remaining 84 participants were allocated to the intervention group (*n* = 44) and the control group (*n* = 40) (Fig. 1). Of the 84 participants, 71 (85%) completed the follow-up assessment, including 38 (86%) in the intervention group and 33 (83%) in the control group, resulting in a dropout rate of 15%. The reasons for dropping out include transportation challenges, health issues, family care obligations, and community commitment.

Among the participants who completed the study, the overall exercise class attendance rate was 95%, with 95% for the intervention group (97% for the Tai Chi sessions, 93% for the resistance-training sessions) and 94% attendance for the control group (94% for the resistance-training sessions). In the intervention group, 34 participants (90%) adhered to the Tai Chi exercise program and 35 (92%) adhered to the resistance-training program (attendance rate ≥ 80%). In the control group, 32 participants (97%) adhered to the resistance-training program (attendance rate ≥ 80%). Common reasons for absences were fatigue, family commitments, community commitments, doctor appointments, health-related issues, vacations, and time conflict with work. During the study, there were no major or minor adverse events that required medical attention.

The characteristics of the study participants are summarized in Table 1. Among those who completed the study, there were no significant between-group differences at baseline in age, sex, educational level, BMI, comorbidities, current tobacco consumption, current alcohol consumption, fall history, pain intensity, number of pain sites, or pain-related disability. Of all participants, 54 (76%) had multisite pain (number of pain sites ≥ 2), with 29 (76%) in the intervention group and 25 (76%) in the control group. The most frequently reported painful sites were shoulders (31%), low back (30%), knees (25%) and other places including hands, feet, and hips (14%).

**Table 1:** Baseline characteristics of the study participants

	Intervention group (*n* = 38)	Control group (*n* = 33)	*p*-value
Age, years	74.29 ± 3.69	73.67 ± 4.49	0.52
Women, n (%)	25 (65.8)	25 (75.8)	0.36
Education level, < 10 years, n (%)	4 (10.5)	0 (0)	0.06
BMI, kg/m^2^	23.84 ± 3.49	22.46 ± 2.93	0.08
Comorbidities, n (%)	33 (86.8)	30 (90.9)	0.59
Current tobacco consumption, n (%)	0 (0)	1 (3.0)	0.28
Current alcohol consumption, n (%)	16 (42.1)	15 (45.5)	0.78
Fell in the past year, n (%)	6 (15.8)	4 (12.1)	0.66
Pain intensity	4.11 ± 1.96	3.94 ± 2.11	0.73
Number of pain sites	2.00 (1.75, 3.00)	2.00 (1.00, 3.00)	0.59
Pain-related disability, points	6.00 (2.75, 8.50)	5.00 (1.50, 7.50)	0.39

Note: Data are presented as mean ± SD, median (interquartile range) or n (%)

*BMI* Body mass index

**Fig. 2:**
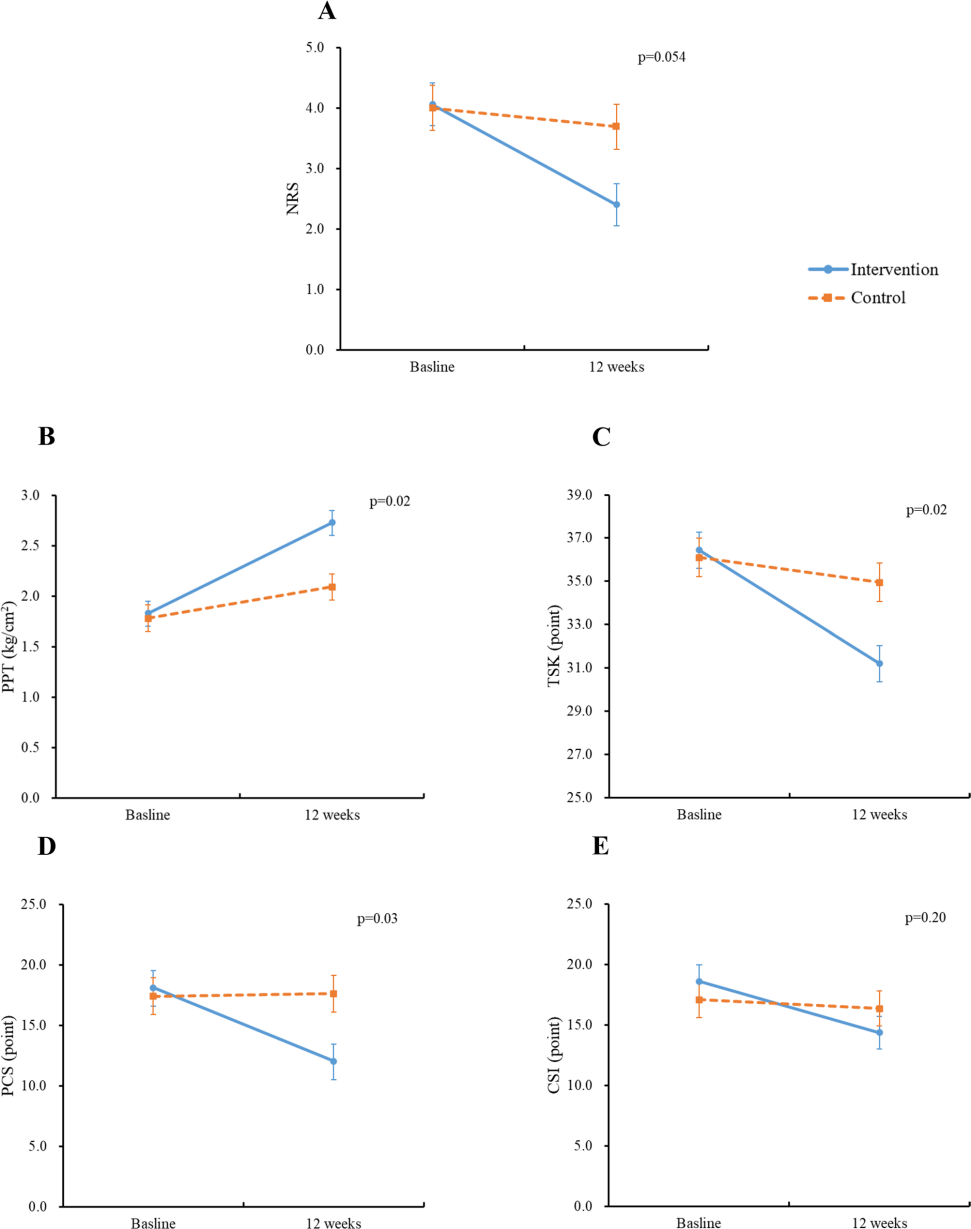
Interaction effect between group and time in pain-related outcomes. **A** NRS (numeric rating scale), **B** PPT (pressure pain threshold), **C** TSK (Tampa Scale of Kinesiophobia), **D** PCS (Pain Catastrophizing Scale), and **E** CSI (Central Sensitization Inventory). *Solid line*: the intervention group. *Dashed line*: the control group. P-values represent the results of the interactions between time and group, adjusting for age, sex, education, BMI, comorbidities, current tobacco consumption, current alcohol consumption, and fall history

Table 2 and Fig. 2 present the changes in pain-related outcomes from baseline to 12 weeks in the two groups. At 12 weeks, the PPT values in both the intervention group and the control group were significantly improved compared to the baseline values. The intervention group’s NRS, TSK, PCS, and CSI scores were significantly decreased at 12 weeks, whereas there was no significant improvement in the control group. Compared to the control group, the intervention group showed significant improvements on the NRS (mean difference = − 1.19, 95%CI: −2.20, − 0.18, *p* = 0.02; d = 0.55, MCID 1.01), PPT (mean difference = 0.61, 95%CI: 0.22, 0.99, *p* = 0.003; d = 0.74, MCID 0.31), TSK (mean difference = − 3.79, 95%CI: −6.35, − 1.22, *p* = 0.004; d = 0.71, MCID 2.60), and PCS (mean difference = − 5.81, 95%CI: −10.18, − 1.43, *p* = 0.01; d = 0.62, MCID 4.13), while there was no significant between-group difference in the CSI score (mean difference = − 2.59, 95%CI: −6.50, − 1.33, *p* = 0.21; d = 0.31, MCID 3.96) (Table 2). After adjustment for age, sex, education, BMI, comorbidities, current tobacco consumption, current alcohol consumption, and fall history, there were still significant interactions for the PPT (*p* = 0.02), TSK (*p* = 0.02), and PCS (*p* = 0.03), and a marginal interaction for the NRS (*p* = 0.054), but no interaction for the CSI (Fig. 2).

**Table 2:** Changes from baseline to 12 weeks in the intervention and control groups for pain-related outcomes

Variable	Study group		Between-group difference (95%CI)	
	Intervention (*n* = 38)	Control (*n* = 33)	Intervention vs. Control	*p*-value
NRS				
Baseline	4.11 ± 1.96	3.94 ± 2.11	0.17 (− 0.80 to 1.13)	0.73
12 weeks	2.45 ± 1.87**	3.64 ± 2.41	−1.19 (− 2.20 to − 0.18)	0.02
PPT				
Baseline	1.81 ± 0.68	1.80 ± 0.52	0.01 (− 0.28 to 0.30)	0.96
12 weeks	2.71 ± 0.87**	2.11 ± 0.75*	0.61 (0.22 to 0.99)	0.003
TSK				
Baseline	36.42 ± 5.82	36.09 ± 4.51	0.33 (− 2.16 to 2.82)	0.79
12 weeks	31.18 ± 5.90**	34.97 ± 4.75	−3.79 (− 6.35 to − 1.22)	0.004
PCS				
Baseline	18.03 ± 7.46	17.52 ± 9.18	0.51 (− 3.43 to 4.45)	0.80
12 weeks	11.92 ± 7.46**	17.73 ± 10.89	−5.81 (− 10.18 to − 1.43)	0.01
CSI				
Baseline	18.32 ± 7.24	17.39 ± 8.72	0.92 (− 2.86 to 4.70)	0.63
12 weeks	14.08 ± 6.02**	16.67 ± 10.23	−2.59 (− 6.50 to 1.33)	0.21

*CI* Confidence interval, *CSI* Central Sensitization Inventory, *NRS* Numeric Rating Scale, *PPT* Pressure pain threshold, *PCS* Pain Catastrophizing Scale, *TSK* Tampa Scale of Kinesiophobia

Note: *p < 0.05, **p < 0.01 paired t-test comparing post- to pre-intervention measures within each group

**Fig. 3:**
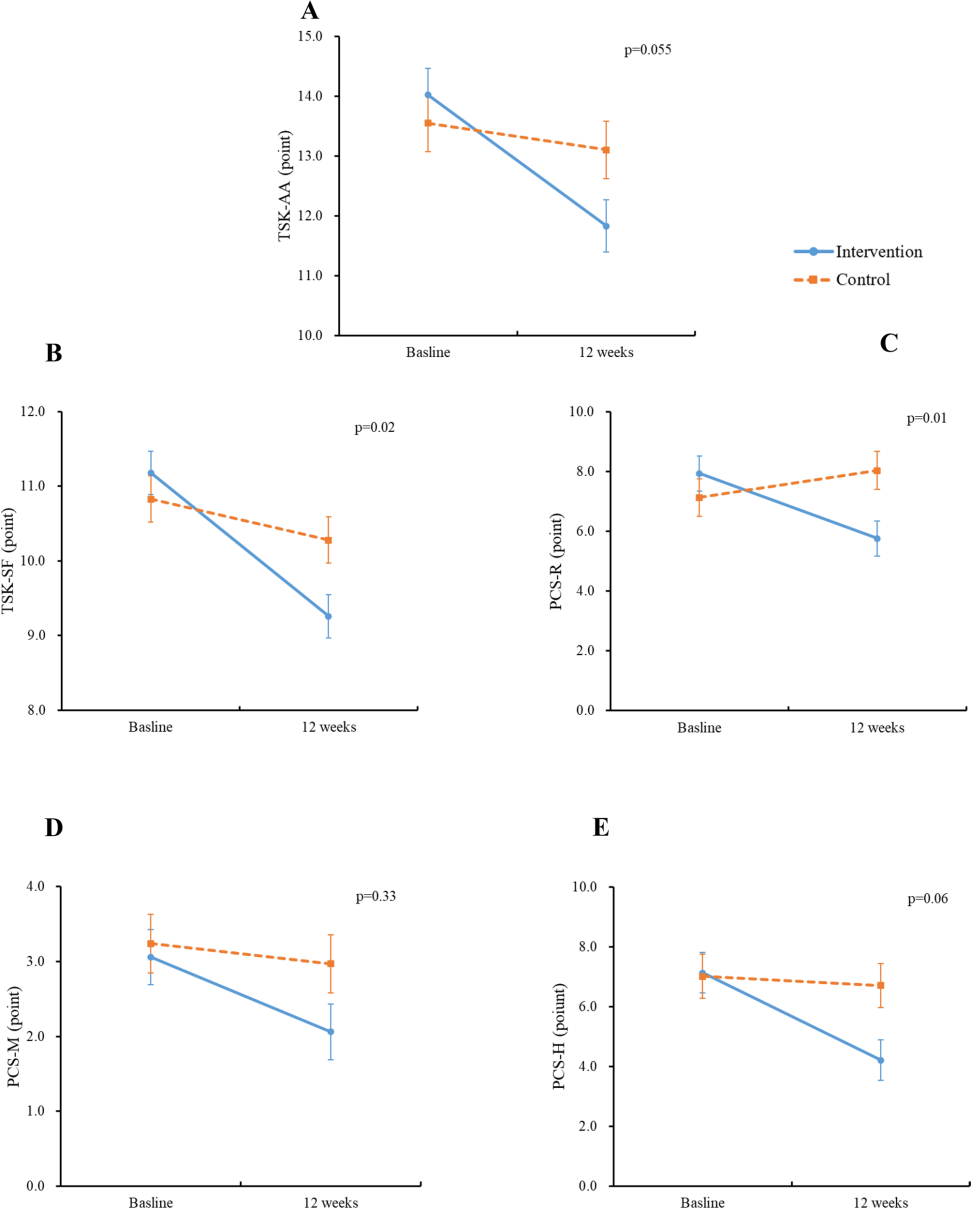
Interaction effect between group and time in subscales of TSK and PCS. **A** TSK-AA (Tampa Scale of Kinesiophobia-Activity Avoidance), **B** TSK-SF (Tampa Scale of Kinesiophobia-Somatic Focus), **C** PCS-R (Pain Catastrophizing Scale-Rumination), **D** PCS-M (Pain Catastrophizing Scale-Magnification), and **E** PCS-H (Pain Catastrophizing Scale-Helplessness). *Solid line*: the intervention group. *Dashed line*: the control group. P-values represent the results of the interactions between time and group, adjusting for age, sex, education, BMI, comorbidities, current tobacco consumption, current alcohol consumption, and fall history

Table 3 and Fig. 3 concern the changes from baseline to 12 weeks in the subscales of the TSK and PCS. At 12 weeks, the intervention group’s TSK-AA, TSK-SF, PCS-R, PCS-M, and PSC-H scores were significantly improved; there were no significant improvements in these scores in the control group. Compared to the control group, the intervention group showed significant improvements in the TSK-SF (mean difference = − 1.07, 95%CI: −1.93, − 0.20, *p* = 0.02; d = 0.58, MCID 0.90), PCS-R (mean difference = − 2.72, 95%CI: −4.44, − 1.00, *p* = 0.002; d = 0.74, MCID 1.79) and PCS-H (mean difference = − 2.37, 95%CI: −4.51, − 0.24, *p* = 0.03; d = 0.53, MCID 1.91), while there was no significant between-group difference in the TSK-AA (mean difference = − 1.31, 95%CI: −2.70, 0.09, *p* = 0.07; d = 0.44, MCID 1.34) or PCS-M (mean difference = − 0.80, 95%CI: −1.94, 0.33, *p* = 0.03; d = 0.18, MCID 1.07) (Table 3). After adjustment for age, sex, education, BMI, comorbidities, current tobacco consumption, current alcohol consumption, and fall history, there was still a significant interaction for the TSK-SF (*p* = 0.02), PCS-R (*p* = 0.01) and a marginal interaction for the TSK-AA (*p* = 0.055), but no interaction for the PCS-M or PCS-H (Fig. 3).

**Table 3:** Changes from baseline to 12 weeks in the intervention and control groups for subscales of the TSK and PCS

Variable	Study group		Between-group difference (95%CI)		
	Intervention (*n* = 38)	Control (*n* = 33)	Intervention vs. Control	*p*-value	
TSK-AA					
Baseline	14.00 ± 2.72	13.58 ± 2.66	0.42 (− 0.85 to 1.70)	0.51	
12 weeks	11.82 ± 3.09**	13.12 ± 2.77	−1.31 (− 2.70 to 0.09)	0.07	
TSK-SF					
Baseline	11.16 ± 1.87	10.86 ± 1.70	0.31 (− 0.54 to 1.16)	0.47	
12 weeks	9.24 ± 1.87**	10.30 ± 1.78	−1.07 (− 1.93 to − 0.20)	0.02	
PCS-R					
Baseline	7.74 ± 3.26	7.36 ± 3.93	0.37 (− 1.33 to 2.08)	0.66	
12 weeks	5.55 ± 3.04**	8.27 ± 4.19	−2.72 (− 4.44 to − 1.00)	0.002	
PCS-M					
Baseline	3.11 ± 1.97	3.18 ± 2.35	−0.08 (− 1.10 to 0.95)	0.88	
12 weeks	2.11 ± 1.86**	2.91 ± 2.89	−0.80 (− 1.94 to 0.33)	0.18	
PCS-H					
Baseline	7.18 ± 3.78	6.94 ± 3.91	0.25 (− 1.58 to 2.07)	0.79	
12 weeks	4.26 ± 3.82**	6.64 ± 5.18	−2.37 (− 4.51 to − 0.24)	0.03	

*CI* Confidence interval, *PCS-H* Pain Catastrophizing Scale-Helplessness, *PCS-M* Pain Catastrophizing Scale-Magnification, *PCS-R* Pain Catastrophizing Scale-Rumination, *TSK-AA* Tampa Scale of Kinesiophobia-Activity Avoidance, *TSK-SF* Tampa Scale of Kinesiophobia-Somatic Focus

Note: *p < 0.05, **p < 0.01 paired t-test comparing post- to pre-intervention measures within each group

The mean change from baseline to 12 weeks in the intervention group exceeded the MCID for the following outcomes: the NRS, PPT, TSK, PCS, CSI, TSK-AA, TSK-SF, PCS-R, and PCS-H. However, the mean change for the PCS-M did not surpass the corresponding MCID. In contrast, no outcomes in the control group exceeded the MCIDs.

## Discussion

To our knowledge, this is the first study to examine the effects of Tai Chi on chronic pain in Japanese community-dwelling older adults. The results demonstrate that Tai Chi is both feasible and acceptable for this population, with retention and adherence rates exceeding our expectations. The study’s primary findings indicate significant interactions between time and group for the PPT, TSK, and PCS, as well as for the subscales TSK-SF and PCS-R, after the adjustment for the participants’ baseline characteristics. The significant data that we obtained suggest that Tai Chi may effectively address both pain symptoms and psychological factors associated with chronic pain.

Moreover, the intervention group demonstrated clinically meaningful improvements in the PPT, TSK, and PCS, with these outcomes exceeding the MCIDs, further supporting the clinical significance of the Tai Chi intervention. Although the mean change in the intervention group’s CSI score surpassed the MCID, the small effect size and lack of a significant interaction suggest that Tai Chi may have a more limited impact on central sensitization components. However, the overall findings emphasize Tai Chi’s potential as a multidimensional intervention for the management of chronic pain.

### Effectiveness of Tai Chi for relieving pain

In a similar study of a community-based population, it was discovered that Tai Chi led to greater improvements in chronic pain symptoms (including pain severity and pain interference) whereas light physical exercise did not improve chronic pain symptoms in older adults with multisite pain [19]. Aligning with that report, our present investigation revealed that the combination of Tai Chi and resistance training led to a reduction in pain severity, whereas resistance training alone did not reduce pain intensity. Significant between-group statistical differences in these changes were observed after the intervention, with a marginal interaction between time and group for pain intensity. These findings further support the efficacy of Tai Chi in improving chronic pain symptoms.

### Effects on pain sensitivity

A systematic review and meta-analysis demonstrated that compared to non-exercise training comparators, exercise may be effective for reducing pain sensitivity (increasing pressure pain thresholds) [32]. Our present results are consistent with this research, as they demonstrated that resistance training can lead to a reduction in pain sensitivity. However, there have been few investigations of changes in pressure pain thresholds after Tai Chi practice. Our study fills this gap and indicates that the combination of Tai Chi and resistance training leads to even greater increases in pain thresholds compared to resistance training alone. We observed significant effects of time and group as well as a significant interaction between time and group, suggesting that Tai Chi also has a pronounced effect on reducing pain sensitivity. Exercise that induces a multi-segmental decrease in pain sensitivity may involve both serotonergic and opioidergic mechanisms [33]. Given its nature as a mind-body exercise, Tai Chi likely plays a significant role in facilitating these mechanisms.

### Effects on kinesiophobia

A recent meta-analysis showed that Tai Chi could alleviate the fear of falling, improve balance, and reduce the fall incidence among older adults [34]. Our study corroborates these findings by showing that Tai Chi could reduce the participants’ fear of movement. Moreover, a new finding from the present study is that Tai Chi had a greater ability to reduce individuals’ somatic focus rather than activity avoidance. This is particularly intriguing given the established associations between somatic focus and negative affect. Although individuals with high levels of somatic focus may indeed experience negative affective states via physical symptoms, it is important to recognize that a somatic focus may serve a dual role in the chronic pain experience. On one hand, excessive focus on pain can exacerbate negative emotions, and on the other hand, patients’ ability to ‘tune in’ to their bodies and accept physical symptoms as they are could potentially be harnessed as a valuable tool in chronic pain management. This aligns with mindfulness principles, where focusing on the “here and now” helps patients distance themselves from past regrets and future anxieties and instead concentrate on their present bodily sensations, enabling them to free themselves from the emotional and cognitive grip of pain, allowing for a more objective and effective way to manage symptoms [35].

The mindfulness practices of Tai Chi can facilitate a transition from a fragmented perception of the body to a unified and interconnected awareness of the body as a whole. This transition enables individuals to be fully present in their physical experiences, allowing them to anticipate and respond to the discomfort of pain more effectively as evidenced by a fieldwork study conducted in Denmark [36]. By promoting a heightened awareness of bodily sensations and fostering a mindful connection between the mind and body, Tai Chi may help individuals shift their focus away from pain and towards a more balanced state of physical and psychological well-being.

### Effects on pain-catastrophizing

Previous research has emphasized the significant role of Tai Chi in reducing catastrophizing, which contributes to the effectiveness of Tai Chi in alleviating pain [37]. Our present findings also indicate that Tai Chi effectively reduced pain-catastrophizing in older adults with chronic pain. Specifically, our interaction results showed that Tai Chi was particularly effective in mitigating the rumination component of pain-catastrophizing, which involves persistent and repetitive thoughts about pain. This reduction in rumination was more pronounced compared to the magnification component (exaggerating the threat of pain) or the helplessness component (feeling powerless to manage pain). These findings suggest that Tai Chi practice may help individuals shift their focus away from pain, thereby fostering a more positive cognitive response.

### Effects on central sensitization

It has been demonstrated that high-intensity cardiorespiratory interval training combined with high-intensity general resistance training can effectively improve symptoms of central sensitization in individuals with chronic nonspecific low back pain, as observed at a 6-month follow-up [38]. In our study, while resistance training alone did not lead to significant changes in CSI, the combination of Tai Chi and resistance training was effective in improving CSI. Despite the absence of a significant between-group difference after the present intervention, the observed improvement in central sensitization symptoms in the intervention group (who performed a combination of Tai Chi and resistance training) indicates a potential benefit of combining these two modalities.

### Possible mechanisms based on these findings

Our findings suggest several interconnected mechanisms by which Tai Chi may contribute to improved pain outcomes in older adults with multisite chronic pain. First, Tai Chi exercises appear to enhance individuals’ cognitive and emotional responses to pain, particularly by reducing catastrophic thinking. This cognitive shift, characterized by a decrease in rumination, is crucial as it directly influences the emotional and psychological dimensions of chronic pain. Secondly, fear of movement and a somatic focus are significant barriers to physical activity in individuals with chronic pain, perpetuating a cycle of inactivity and increased pain perception. By alleviating the fear of movement and reducing the excessive focus on negative emotions and cognitions resulting from pain, Tai Chi encourages greater engagement in physical activities. This is vital for maintaining physical function and overall health, as it helps break the cycle of inactivity and chronic pain. Thirdly, increased exercise and physical activity induce physiological changes such as the release of endogenous opioids and serotonin, which help modulate pain perception and increase the pressure pain threshold. Finally, improved pain thresholds — as a result of the neurophysiological adaptations — could lead to a reduction in pain intensity and the improvement of ADLs.

In summary, the results of this examination of 71 older adults indicate that Tai Chi exercises may improve pain outcomes through a cascade of interconnected effects: enhancing cognitive responses by potentially reducing catastrophic thinking, alleviating fear of movement and somatic focus, and possibly inducing neurophysiological adaptations that could increase pain thresholds. These changes may lead to a reduction in pain intensity. This holistic approach, which addresses both the physiological and psychological aspects of chronic pain, suggests the importance of treating the mind and body in unison for effective chronic pain management.

### Study strengths and limitations

Our participants achieved a high adherence rate, which was facilitated by proactive measures such as encouragement from the exercise and Tai Chi instructors during the sessions and reminder phone calls. This surpasses adherence rates observed in similar community-based studies, possibly attributed to cultural or demographic variations [19], which enhances the reliability of our findings. Our results demonstrated Tai Chi’s impact on pain sensitivity, kinesiophobia, pain-catastrophizing, and central sensitization, which adds to the comprehensive mechanistic understanding of how Tai Chi achieves behavioral changes and pain-relief effects.

Understanding patients’ levels of somatic focus and rumination may assist providers in interpreting symptom reports and conceptualizing chronic pain more effectively. Accordingly, more effective implementation of treatment efforts could be aided by the assessment of patients’ somatic focus and rumination. This knowledge can facilitate more effective implementation of treatment efforts, such as educating patients about the mind-body connection to better manage negative moods and pain and encouraging their participation in non-pharmacological pain treatments.

Our study has several limitations. First, the lack of randomization introduces the possibility of selection bias that could potentially influence the outcomes, as the participants voluntarily chose to participate in the resistance and Tai Chi sessions. Second, the small sample size (*n* = 71) and single-center design limit the generalizability of the findings to broader populations. Although the results may show some clinical significance, the relatively small sample size reduces the robustness of our statistical comparisons and highlights the need for a cautious interpretation of the study’s findings. Third, our participant group, which did not exceed the cutoff values for the questionnaires and maintained relatively good physical function, represents a relatively healthy population. This limits the generalizability of our findings to individuals with more severe pain-related impairments. In addition, potential confounding factors such as medication use, pain characteristics beyond pain intensity, and the number of pain sites were not systematically addressed in this study. Although none of the baseline characteristics differed significantly between the intervention and control groups, reducing the risk of confounding, this limitation cannot be entirely ruled out. Moreover, the slightly lower adherence rate in the intervention group compared to the control group may have contributed to an overestimation of the effect of Tai Chi. Despite these limitations, the sensory-emotional-cognitive effects observed suggest that Tai Chi may provide significant benefits among community-dwelling older adults with chronic pain. Last, the relatively short duration of this study’s intervention and follow-up period may not capture long-term effects or the sustainability of the intervention. These limitations underscore the need for larger, randomized controlled trials with extended follow-up periods to validate the findings.

This study demonstrated Tai Chi’s positive impacts on sensory-emotional-cognitive aspects, but it is important to recognize that the benefits of Tai Chi in reducing pain symptoms could stem from other complex interactions. For example, a systematic review concluded, based on two non-randomized studies, that there was preliminary evidence of brain adaptation with exercise in people with pain syndromes, implying that Tai Chi may elicit similar neuroadaptive responses [39]. Besides, it is worth noting that the breathing techniques in Tai Chi may also have potential effects on the autonomic nervous system (ANS) [40]. The ANS plays a crucial role in pain mechanisms, interacting with both peripheral and central pathways and potentially influencing pain perception and modulation [41]. To comprehensively grasp Tai Chi’s efficacy, future investigations should delve into the interplay of biomechanical, biochemical, neurological, and social factors in order to elucidate Tai Chi’s holistic impact on pain management [42, 43].

## Conclusions

The results obtained in this study indicate that the 12-week Tai Chi intervention was well-received and feasible among Japanese community-dwelling older adults with chronic pain. While our findings indicate a potential for Tai Chi to alleviate chronic pain by addressing various sensory, emotional, and cognitive components of chronic pain, the evidence is preliminary and warrants further investigation. Future research utilizing more robust experimental designs, larger cohorts, and extended follow-up periods is necessary to confirm the clinical efficacy of Tai Chi and to further elucidate its underlying therapeutic mechanisms.

## Data Availability

No datasets were generated or analysed during the current study.
